# Varied Growth Media Necessitate Different Light Regimes for Indoor Duckweed Cultivation

**DOI:** 10.3390/plants14030397

**Published:** 2025-01-28

**Authors:** Cian Redmond, Rachel O’Mahoney, Marion Blanchard, Neil E. Coughlan

**Affiliations:** School of Biological, Earth and Environmental Sciences & Environmental Research Institute, University College Cork, T23 TK30 Cork, Ireland; credmond@ucc.ie (C.R.);

**Keywords:** agricultural wastewater, circular economy, daily light integral, Lemnaceae, light intensity, photoperiod

## Abstract

Controlled indoor cultivation of duckweed plants can support remediation of wastewaters through generation of plant biomass. Despite numerous advantages, indoor cultivation of duckweeds on agri-food wastewaters remains underexplored. Lighting regimes need to be optimised for duckweed growth and affordability of energy consumption, as it has been shown that the composition of wastewater growth medium can alter light utilisation. In the present study, four duckweed (*Lemna minor*) clones were grown under four different light regimes on either optimised half-strength Hutner’s medium or wastewater derived from the liquid fractions of anaerobically digested pig slurry. Cultivation of *L. minor* was assessed for the four light regimes using a commercial hydroponics plant growth medium in a 3.96 m^2^ multitiered cultivation system. When cultivated on optimised half-strength Hutner’s medium or diluted pig slurry under laboratory conditions, it appeared that photoperiod rather than light intensity was more important for duckweed growth. Yet, under moderate flow conditions within a larger scale multitiered cultivation system, greater light intensity appeared to support duckweed cultivation irrespective of photoperiod. These findings emphasise the need to move beyond small-scale and static assessments of duckweed before embarking on larger, industry-relevant scales.

## 1. Introduction

Livestock production along with food processing generates large volumes of numerous liquid waste streams frequently enriched with excess nutrients, such as nitrogen and phosphorus [1]. If discharged without sufficient treatment, these wastewaters can pose a serious threat to water quality, human health, and the function of aquatic ecosystems through eutrophication [2]. In many cases, the need to comply with wastewater treatment regulations tends to be operationally burdensome and economically costly for the wider agri-food sector [3]. Typical approaches for removal of excess nutrients involve microbial degradation of the available organic carbon, coupled with the oxidation of ammonia to nitrate followed by denitrification and chemical precipitation of phosphates [4]. Wastewater treatment is a considerable financial cost, without the generation of direct benefits to business enterprises. Moreover, wastewater volumes are expected to increase alongside the predicted continued expansion of the agri-food sector [5]. Accordingly, the adoption of circular economy principles is increasingly considered as an imperative step towards the long-term environmental and economic sustainability of the agri-food sector. In brief, circular economy principles seek to reduce demand for new inputs, such as raw resources and fossil fuels, and promote the use of wastes and byproducts as resources for further exploitation through their retention in energy and production cycles [6]. The application of circular economy principles in management of agri-food wastewaters remains a considerable challenge, with a clear need for the development of innovative wastewater treatment approaches that can facilitate valorisation through remediation and recovery of valuable nutrients.

Duckweeds are small aquatic plants that float on or near the surface of water. Duckweeds rapidly reproduce asexually through clonal budding, typically displaying rapid growth rates with a biomass doubling time of 2–3 days under optimal conditions [7], along with an ability to uptake large quantities of nitrogen and phosphorus [8,9] and metals [10]. Duckweed species tend to have a high protein content, with some species comprising up to 45% dry-weight protein content [11], as well as a favourable amino acid profile that betters that of soybean meal [12,13]. Consequently, duckweed cultivation is increasingly considered a valuable addition to circular economy wastewater treatments, to enable remediation as well as the generation of economically valuable high-protein biomass suitable for use in livestock feeds [14]. Under favourable conditions of sufficient light, nutrients, and temperature, duckweeds can be grown year-round, which has further underpinned scientific and industry interest in use of duckweed in both outdoor, semi-outdoor and controlled indoor cultivation systems. In particular, indoor duckweed cultivation systems have distinct advantages that enable year-round cultivation under controlled environmental conditions, where temperature, humidity, light spectrum, and photoperiod are all optimised [15]. Furthermore, through use of multitiered vertical cultivation systems, indoor cultivation can also be space efficient [16]. These attributes, amongst others, make indoor duckweed cultivation a desirable plant-based treatment approach compared to alternatives such as constructed wetlands that offer little economic incentive with efficacy of remediation restricted to the growing season.

Indoor duckweed cultivation requires the provision of carefully selected lighting conditions to support optimal growth and nutrient recovery. Light conditions also need to be selected to balance energy and cost considerations, infrastructure development, and operation with growth benefits. Optimisation of lighting conditions, to maximum biomass production and nutrient removal from a wastewater while conserving energy and limiting running costs, is an essential yet poorly considered aspect of larger-scale indoor duckweed cultivation [15]. The approach taken will likely vary across different industries operating within the wider agri-food sector, as inter alia physicochemical properties of wastewaters can vary considerably between different agri-food enterprises and thus can require varied management practices with tailored remediation. Duckweed clones (or “ecotypes”) of a single species can display differing tolerances based on localised exposure to different environmental conditions, which likely results in some clones being more suitable for cultivation on wastewaters [17].

Previous research has shown that the optimal light intensity for duckweed growth varies based on the species of duckweed and physicochemical properties of the growth medium, with growth rates on some wastewaters saturating at relatively low light intensities [18,19]. It has been demonstrated for some common horticultural crops that longer photoperiods with lower light intensity can result in faster plant growth compared to shorter photoperiods with higher light intensity, with the same daily light integrals (DLIs) (e.g., [20]). However, in the case of continuous light, the long photoperiod can either increase or decrease plant development, with outcomes appearing to depend on a host of abiotic factors (e.g., temperature, light intensity, CO_2_ level, nutrient availability; Sysoeva et al., 2010). Continuous light may lead to increased photooxidation that over time leads to chlorosis, as well as interfering with circadian rhythms [21]. Landolt and Kandeler [22] reported that highest growth rates of Lemnaceae occurred under continuous light, although the combination of continuous light at high intensity can be damaging. More recent studies have either confirmed the growth enhancement [23] or found no notable effect [24]. Yet, little is known about the effects of different photoperiods and light intensities on the cumulative biomass yield and composition, and remediation capacity of duckweed, when cultivated on optimal and sub-optimal media.

Traditional plant protein crops (i.e., soybean) are relatively inexpensive to scale but come with several disadvantages that can offset their benefits. Firstly, land-use efficiency is low, as these crops require vast areas to grow [25]. Secondly, they depend heavily on inputs such as irrigation and synthetic fertilisers, which not only increase costs for farmers but also create environmental challenges, including soil salinisation, erosion, and nutrient runoff, which can lead to the eutrophication of waterways [26]. Furthermore, traditional crops are seasonal and subject to weather conditions, making them vulnerable to adverse weather events, pests, and pathogens. Additionally, the proteinaceous element of many crops represents only a small fraction of the plant, leaving crop residue with little value [27]. Duckweed-based protein production offers a solution to many of these challenges. Indoor duckweed cultivation is highly space-efficient, as several square meters of duckweed can be grown on a single square meter of land [16]. Water and nutrient use efficiency are greatly improved and can be precisely controlled to minimise waste [15]. Unlike outdoor crops, indoor duckweed systems are not weather-dependent and can operate year-round, offering consistent production regardless of the season. Additionally, the entire plant can be harvested as needed, reducing waste and maximising yield. In the case of the duckweed biomass being used to produce a protein isolate, any remaining biomass can be utilised for biogas production (i.e., methane) and lactic acid production [28]. Indoor cultivation also allows for more controlled environmental conditions, which can result in high-yielding and quality protein and a greater value per tonne of harvested biomass [29]. Duckweed based systems can be automated, reducing labour costs with technologies such as automatic harvesting arms and nutrient dosing pumps [15,16]. While indoor systems require supplementary light, the increasing affordability of solar, wind, and biogas energy makes this a viable and sustainable option [30].

The present study assessed the cultivation of four different *L. minor* clones under four light regimes. Growth of four selected *L. minor* clones was assessed using either a laboratory-grade nutrient-rich medium (i.e., half-strength Hutner’s), or wastewater derived from the liquid fractions of anaerobically digested pig slurry obtained through vibratory shear enhanced processing (VSEP ADLF-pig slurry). Intensive livestock production has led to challenges in the management, treatment, and application of manures, with solid–liquid separation increasingly used to improve the agronomic characteristics of semi-solid wastes into concentrated solid manure, but simultaneously results in less concentrated and economically burdensome liquid fractions (e.g., [31]). Lastly, larger-scale cultivation of one *L. minor* clone was assessed under the four selected light regimes using a commercial-grade hydroponics medium within a multitiered, indoor duckweed cultivation system. A higher DLI was expected to support improved growth on half-strength Hutner’s medium. However, it was anticipated that this would not be the case for clones cultivated on wastewater. Rather, given that low light intensity has previously been observed to support optimal growth for duckweed cultured on sub-optimal media (e.g., [19]), it was expected that low light would underpin improved growth.

## 2. Methods

### 2.1. Light Regimes

Light regimes consisted of four light intensities (35, 55, 60, and 100 µmol m^−2^ s^−1^) provided at photoperiods of either 16 h light and 8 h dark (16:8) or 24 h light and 0 h dark (24:0). Light intensities and photoperiods were selected to facilitate similar DLIs between the two high intensities and separately between the low intensities (see Table 1).

### 2.2. Experiment 1: Stock Cultivation

The duckweed clones used for Experiment 1 included *Lemna minor* L. “Blarney”, number 5500 in the Rutgers Duckweed Stock Cooperative database, as well as three additional clones collected from throughout southwest Ireland: “Ballinacurra”, “Mitchelstown”, and “Sherkin” (Table 2). Clones collected from the wild were identified as *L. minor* based on morphological characteristics [32]. All source locations were separated by linear distances of between 40 and 120 km.

*Lemna minor* “Blarney” was sourced from axenic stocks cultured at the School of Biological Earth and Environmental Sciences (BEES), University College Cork, Ireland. Specimens of the three other clones, collected from the field, were transported to the School of BEES, University College Cork, Ireland on source water. These specimens were allowed to acclimate to laboratory conditions for about 3–5 days on source water before being transferred to half-strength Hutner’s medium, an optimised growth medium for duckweed cultivation [33]. Non-axenic stock cultures of all *L. minor* specimens were maintained indoors in optimised growing conditions on half-strength Hutner’s medium within a growth room at a constant temperature of 22 ± 2 °C, at a light intensity of 50 μmol m^−2^ s^−1^ PAR (photosynthetically active radiation) provided by cool white fluorescence tubes, with a 16:8 h light/dark photoperiod. Non-axenic cultures were maintained for 2–3 weeks before experimentation.

### 2.3. VSEP ADLF-Pig Slurry Physicochemical Analysis

The liquid fractions of anaerobically digested pig slurry (ADLF-pig slurry) obtained from vibratory shear enhanced processing (VSEP), used for separation of solids and liquids, was sourced from a medium-scale commercial pig farm. A full physicochemical assessment was conducted on the ADLF-pig slurry effluent released from VSEP separation. Non-metal characteristics were analysed by an INAB EN ISO/IEC 17025-certified laboratory (ALS Life Sciences Ltd., Clonmel, Co., Tipperary, Ireland), while dissolved metals and major cations were assessed by ČSN EN ISO 14001 (ALS Czech Republic, s.r.o, Vysocany, Prague, Czech Republic). Biochemical oxygen demand (BOD), chemical oxygen demand (COD), total solids, total nitrogen (TN), and total phosphorus (TP) were measured on whole, unfiltered wastewater samples, as per standard methods for wastewater analysis [34,35]. ADLF-pig slurry effluent was filtered (0.45 μm) to determine the dissolved concentrations of ammonia, nitrate, nitrite, and orthophosphate using the Lachat Quik-Chem 8000 by Zeilweger Analytics, Inc., Milwaukee, WI, USA (QuikChem Methods 10-107-06-3-D, 10-107-04-1-C, 10-107-04-1-C, and 10-115-01-1-B, respectively). Sodium, potassium, calcium, magnesium, zinc, iron, copper, and manganese were measured in filtered wastewater (0.45 μm) through the determination of elements by mass spectrometry with inductively coupled plasma and stoichiometric calculations of compounds concentration from measured values including the calculation of total mineralization and calculating the sum of Ca + Mg (W-METMSFL6 method: US EPA 200.8, CSN EN ISO 17294-2, US EPA 6020A, and CSN 75 7358). Chloride was measured using the ferricyanide method on filtered (0.45 µm) wastewater [34]. See Table 3 for results of the full physicochemical analysis. The VSEP ADLF-pig slurry was stored in a controlled temperature room (4 °C) prior to experimental use without any further treatments or amendments following VSEP separation.

### 2.4. Experiment 1: Cultivation of Lemna Minor Clones Under Different Light Regimes

**Figure 1:**
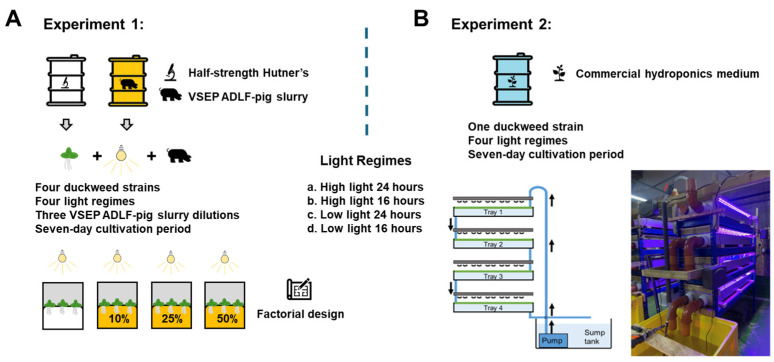
Graphical overview of Experiment 1 (**A**) and Experiment 2 (**B**), with illustration of the multitiered cultivation system. See Coughlan et al. [16] for a detailed description of system design and operation.

The experiment was undertaken using Magenta vessels (Magenta GA-7 Plant Culture Box) and lasted for seven days (Figure 1A). Magenta vessel lids had a central circular hole (~15 mm diameter) that was loosely packed with cotton wool to allow for some gaseous exchange. Duckweed clones were cultivated on half-strength Hutner’s medium. Four different light regimes were assessed, comprising of either a high or low daily light integral (DLI: mol m^−2^ d^−1^), which was achieved using different combinations of light intensity (µmol m^−2^ s^−1^ in the PAR range) and photoperiod (hours of light per day: see Table 1). DLI was calculated by multiplying the mean light intensity for each treatment (Photosynthetic Photon Flux Density in µmol m^−2^ s^−1^) by the photoperiod in seconds and then dividing the result by 1.0 × 10^6^ [38].

Based on the results of the VSEP ADLF-pig slurry physicochemical analysis (see Table 3), cultivation of duckweed clones was assessed for three different dilutions of VSEP ADLF-pig slurry (10%, 25%, and 50% samples). Assessment of a 100% (i.e., non-diluted) sample was omitted, as preliminary data indicated that 100% VSEP ADLF-pig slurry was unsuitable for duckweed growth. All dilutions were created using distilled water and assessed under the four selected light regimes (Table 1).

The experiment commenced on Day 0 with three four-frond colonies taken from the stock culture and placed in each replicate on 250 mL VSEP ADLF-pig slurry dilutions. In a fully factorial experiment, duckweed clones (“Ballinacurra”, “Blarney”, “Mitchelstown” or “Sherkin”) were separately assessed in individual Magenta vessels. Open-top Magenta vessels were placed in a random block design on Day 0, with each treatment replicated in triplicate (i.e., *n* = 3) at a temperature of 22 ± 2 °C. To determine initial fresh biomass, *L. minor* was patted dry using absorbent paper towel and then weighed using a fine balance (Fisherbrand Analytical Series). On the final day of experiments (Day 7) *L. minor* was weighed using the same process for measuring initial biomass, with colony and frond count recorded.

### 2.5. RGR Calculation

Relative growth rate (*RGR*) was calculated using fresh biomass measurements with the following equation [39]:

$$RGR=\frac{\ln\left(\frac{W_{2}}{W_{1}}\right)}{\Delta T}$$

where *ln* is the natural logarithm, *W*_1_ is the initial fresh weight (Day 0), *W*_2_ is the final fresh weight (Day 7), and Δ*T* is the duration of the experiment (7 days).

### 2.6. Experiment 2: Duckweed Multitiered Bioreactor Description

The design and operation of a multitiered, indoor duckweed cultivation system used by the present study is described in detail by Coughlan et al. [16]. Three independent recirculatory bioreactors were used to support replicated testing. In brief, each bioreactor consisted of four vertically stacked stainless-steel cultivation trays (180 cm × 55 cm × 15 cm: length × width × height) suspended within a powder coated steel frame, alongside a sump tank (120 cm × 80 cm × 75 cm) positioned at floor level. Each tray had a total surface area of 0.99 m^2^, giving a total surface area of 3.96 m^2^ in each system. Each duckweed bioreactor was operated as an independent recirculating system with a total medium volume of 500 L. Medium was delivered from the sump tank to the top tray (i.e., tray 1) (Reef Pump 4000 DC Aquarium Pump, Tropical Marine Centre, UK). All three bioreactors were operated at a tray medium depth of 50 mm, with a fixed flow rate from the pump to tray 1 of 2.5 L min^−1^ and then with the force of gravity delivering medium down through the multitiered system through each tray until the medium was returned to the sump tank from tray 4.

All four trays had an identical lighting system. Each comprised a 21 m strip of light-emitting diodes (LEDs) in the PAR range (400–700 nm: Neonica Growy, Neonica Polska Sp. z o.o., Łódź, Poland). Although recent research indicates spectrum may not make a substantial difference for duckweed growth [40], a combination of red, blue, and white LEDs was used to generate a spectrum enriched with blue. Each 21 m × 10 mm wide LED strip was uniformly installed as 12 connected rows, and each row was 175 cm in length with a gap of 30 mm between rows to ensure even distribution of light (±10%), as confirmed using a PAR meter (Skye Instruments Ltd., Powys, UK). The LEDs were suspended 70 mm above the cultivation tray. Accordingly, when operated at a tray medium depth of 50 mm, the LEDs were positioned at 170 mm above the duckweed. The average light intensity could be varied between 0 and 150 μmol m^−2^ s^−1^ PAR using LED dimmer switches. Timers were used to switch LEDs on, controlling the photoperiod as required.

### 2.7. Experiment 2: Stock Cultivation

Non-axenic stock cultures of *Lemna minor* “Blarney” duckweed were cultivated indoors in six open-air tanks (60 cm × 40 cm × 42 cm: length × width × height). Duckweed was maintained on 60 L of a hydroponics medium that consisted of tap water and a two-part commercial liquid fertiliser: pH Perfect Grow (0.25 mL L^−1^) and pH Perfect Micro (0.25 mL L^−1^: Advanced Nutrients). Duckweed was kept at a light intensity of 150 µmol m^−2^ s^−1^ PAR (LED-based lamps: AP67 R-series, Valoya, Finland) with a 16:8 h light/dark photoperiod, at a constant water temperature of 22 ± 2 °C. Duckweed cultivated in these vessels were mixed regularly to ensure homogeneity across the six tanks. Duckweed extracted from the cultivation tanks for use within the experiment was also evenly split across the six source tanks to ensure an even mix of material.

### 2.8. Experiment 2: Bioreactor Cultivation of Lemna Minor “Blarney” Under Different Light Regimes

Duckweed was cultivated in the indoor multitiered stacked systems under four different light regimes for seven days (see Table 1; Figure 1B). Using a fully factorial approach, this was achieved by manipulating light intensity and the photoperiod with each treatment replicated three times across the three modular multitiered cultivation systems (i.e., *n* = 3). The systems were operated at a volume of 500 L and circulated at a flow rate of 2.5 L min^−1^ with a temperature of 22 ± 2 °C. The medium was as previously described for stock cultivation. In total, 250 g of wet-weight duckweed was added to each tray on Day 0, and represented a ~60% surface area cover in each tray. Earlier studies using imaging software Easy Leaf Area had shown that 250 g (fresh weight) gave 60% surface cover of the 0.99 m^2^ trays [16]. Previous studies have also shown that 60% surface cover is optimal for duckweed growth [41]. In turn, a floating plastic ring was inserted into each tray and tethered to remain stationary at the centre of each tray (ring diameter = 370 mm). Floating rings facilitated sub-sampling of each cultivation tray with 28.4 g duckweed being placed in teach ring and 221.6 g being placed in the tray outside each ring (i.e., 250 g total, with 60% surface cover). A 100 mL medium sample was taken from the sump tank and refrigerated (5 °C) for later assessment of medium nitrogen and phosphorous concentrations. The sump tank was topped-up with tap water on an ad hoc basis every 24–48 h to account for evaporation.

On Day 7, prior to harvesting duckweed, water samples were taken in quick succession from the sump tank, the first tray, and the final tray of each multitiered cultivation system. Medium samples were then refrigerated (5 °C) until analysed for nitrogen and phosphorus concentrations. Following this, duckweed was harvested from each individual tray. Using a plastic sieve (2 mm^2^ mesh), duckweed found inside the floating ring was extracted and weighed. Duckweed was patted dry using absorbent paper towel to remove excess water and then weighed using a fine balance (Fisherbrand Analytical Series). Duckweed RGR was calculated for biomass extracted from the floating rings, using the RGR equation as previously described, and then appropriately scaled to the level of the entire cultivation tray.

### 2.9. Experiment 2: Cultivation Medium Nitrogen and Phosphorous Content

Total nitrogen (TN) and total phosphorous (TP) were determined for medium samples using Hach LCK138 Laton (TN test range: 1–16 mg L^−1^ TN_b_) and LCK348 Phosphate (TP test range: 0.5–5.0 mg L^−1^ PO_4_-P) test kits. For the TN assessment, all samples were diluted by 50% with distilled water to ensure sample concentration was within the test kit range. In both cases, the kit instructions were followed in full, and a Hach Spectrometer (DR 3900) was used to determine sample TN and TP concentration for Day 0 and Day 7 samples. These data were used to determine the net TN and TP depletion for the 500 L systems over the seven-day period. In turn, mean TN and TP removal rates were calculated based on the initial 60% surface cover of the total surface cover available within the bioreactor, yielding 2.376 m^2^ (i.e., 60% of 3.96 m^2^). The calculated nutrient removal rates provide an estimate for the milligrams of N and P removed per m^2^ of *L. minor* per day (mg TN m^−2^ d^−1^; mg TP m^−2^ d^−1^).

### 2.10. Data Analysis

Statistical analyses were conducted using R software (R Core Team 2021; R 4.1.2). All data were assessed for normality of residual distributions (Shapiro–Wilk test: library *psych*) and homoscedasticity of variances (Levene’s test: library *car*). Where data were found to be normally distributed (*p* > 0.05) with homoscedastic residuals (*p* > 0.05); general linear models (LM) were used to analysis differences in RGR and nutrient depletion of the media. Logistic regression in the form of generalised linear models (GLM: *car*) were employed for non-normal data and/or heteroscedastic residuals (*p* < 0.05). Data were analysed in relation to independent variables of 1) medium concentration, 2) light treatment, and 3) clone, with consideration of interactive effects applicable. A stepwise deletion approach was used to remove non-significant terms if required, while overall model significance was determined using likelihood ratio tests in all cases (*lmtest*). Where *p*-values were significant (α < 0.05), a Tukey or Benjamini–Yekutieli adjustment for multiple pairwise comparisons was used for post-hoc analysis where appropriate (*emmeans*).

## 3. Results

### 3.1. VSEP ADLF-Pig Slurry Physicochemical Properties

Physicochemical properties are reported for VSEP ADLF-pig slurry in Table 3. BOD and COD concentrations were low, while most compounds tended to be present in amounts within the optimal range for duckweed growth (Table 3). Chloride, sulphate, and sodium concentrations exceeded the range optimal for duckweed growth, while potassium exceeded the maximum concentration tolerated by ~350 mg L^−1^. This may have resulted in 100% VSEP ADLF-pig slurry being unsuitable for duckweed cultivation as previously mentioned.

### 3.2. Experiment 1: Growth of Lemna Minor Clones Under Different Light Regimes

**Figure 2:**
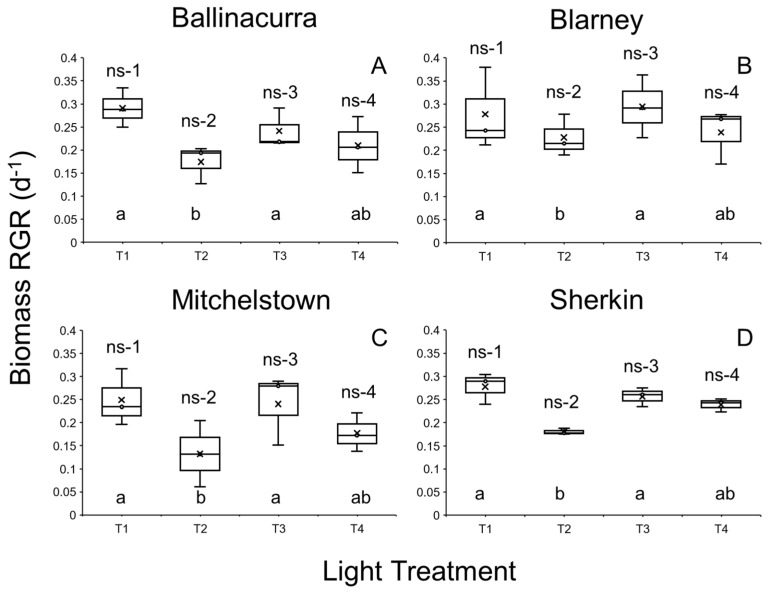
(**A**–**D**) Median relative growth rate for four *Lemna minor* clones grown following exposure to the four different light treatments (T1, T2, T3, and T4; see Table 1) when cultivated on half-strength Hutner’s medium. Interquartile ranges (IQR) and maximum and minimum IQR values are also shown, while × denotes mean values. Clones were collected from different locations throughout southwest Ireland. See Table 1 for description of light treatments. Shared letters indicate statistical similarity among pairwise comparisons within each clone (a or b; *p* > 0.05). Statistical differences were not detected among clones within each light treatment (i.e., no significant difference: ns-1, -2, -3, -4; all *p* > 0.05).

The RGRs of the four selected *L. minor* clones grown on half-strength Hutner’s medium under different light regimes significantly differed (LM: χ^2^ = 30.656, df = 6; *p* < 0.0001; Figure 2). Duckweed clones (*p* < 0.05) and light treatment (*p* < 0.0001) both had a significant effect on RGRs, yet no interactive effect was detected (i.e., *p* > 0.05). For each clone, the RGR of duckweed exposed to the T2 light treatment was significantly lower than that for duckweed exposed to T1 and T3 treatments (all *p* < 0.01). In turn, the RGR of duckweed exposed to the T2 light treatment did not differ from the RGR of duckweed cultivated under the T4 light treatment (all *p* > 0.05; Figure 2). Amongst the assessed clones, the recorded RGRs did not differ within each light treatment (all *p* > 0.05; Figure 2).

**Figure 3:**
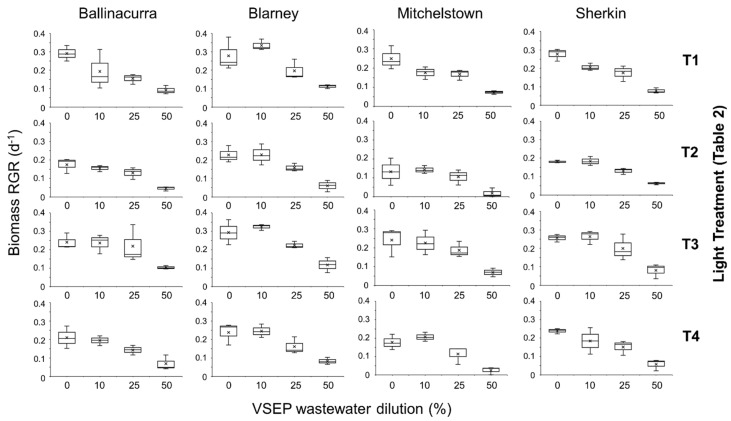
Median relative growth rate for four *Lemna minor* clones grown following exposure to the four different light treatments (T1, T2, T3, and T4; see Table 1). Duckweed was grown on different dilutions of liquid fraction of anaerobically digested pig slurry (ADLF-pig slurry) obtained from the vibratory shear enhanced processing (VSEP). Duckweed grown on half-strength Hutner’s medium were used as a control. Interquartile ranges (IQR) and maximum and minimum IQR values are also shown, while × denotes mean values. Clones were collected from different locations throughout southwest Ireland.

The RGR of selected *L. minor* clones grown in various dilutions of VSEP ADLF-pig slurry under different light treatments significantly differed (LM: χ^2^ = 272.8, df = 9; *p* < 0.0001; Figure 3). Greater dilution tended to increase RGR (*p* < 0.0001; Figure 3), with different RGRs detected among selected *L. minor* clones (*p* < 0.0001), while light regimes also impacted RGR (*p* < 0.0001). No interactive effects were detected (all *p* > 0.05). Overall, when taken as a whole, the RGR of “Blarney” was greater than “Ballinacurra”, “Mitchelstown”, or “Sherkin” (all *p* < 0.001). In turn, duckweed RGR was greater for samples cultivated on a 10% dilution of VSEP ADLF-pig slurry in comparison to duckweed grown on ≥ 25% dilutions (all *p* < 0.0001) but did not differ from control samples (all *p* > 0.05). All light treatments differently impacted RGR (T1:T2, T1:T4, T2:T3, T3:T4 all *p* < 0.001; T2:T4 *p* < 0.05), other than T1:T3 (*p* > 0.05; Figure 3). Trends depicted by pooled data are further supported by a complete matrix of individual pairwise comparisons generated through post-hoc analysis (*n* = 2016; see Supplementary Material).

### 3.3. Experiment 2: Bioreactor Production of Lemna Minor “Blarney” Under Different Light Regimes

**Figure 4:**
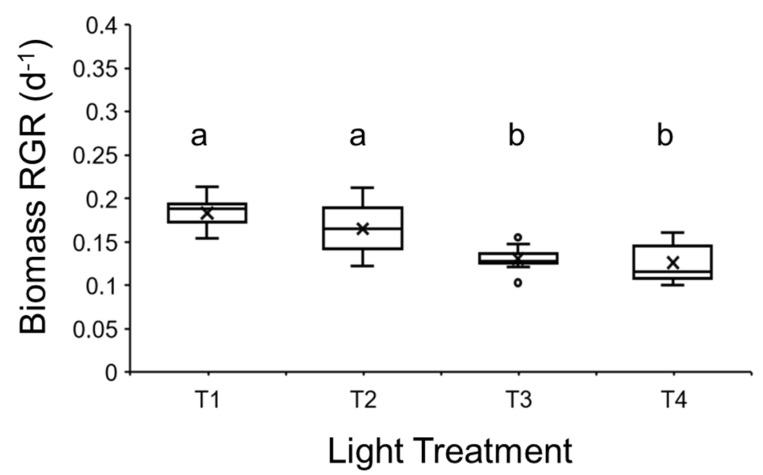
Median relative growth rate for *Lemna minor* “Blarney” clone cultivated within a multitiered duckweed cultivation system under the four different light treatments (T1, T2, T3, and T4; see Table 1). Duckweed was cultivated on a commercial-grade nutrient-rich growth medium. Interquartile ranges (IQR), maximum and minimum IQR values, and outliers (○) are also shown, while × denotes mean values. Shared letters indicate statistical similarity among pairwise comparisons (a or b; *p* > 0.05).

The RGR of *L. minor* grown under different light regimes within the prototype duckweed bioreactor significantly differed (GLM: χ^2^ = 39.924, df = 3; *p* < 0.0001; Figure 4). Duckweed cultivated under T1 and T2 did not statistically differ (*p* > 0.05; Figure 4). In turn, a statistical difference was not apparent between duckweed cultivated under T3 and T4 light treatments (*p* > 0.05). RGR significantly differed between T1:T3 and T1:T4 treatments (both *p* < 0.0001), as did RGR between T2:T3 (*p* < 0.001) and T2:T4 (*p* < 0.0001) (Figure 4).

### 3.4. Experiment 2: Medium Nitrogen and Phosphorous Content

**Figure 5:**
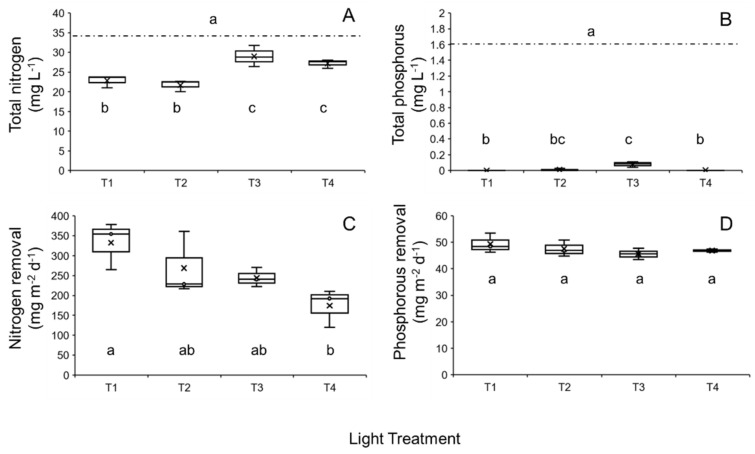
Median total nitrogen (TN: (**A**)) and total phosphorous (TP: (**B**)) content of the commercial-grade growth medium following a 7-day cultivation period for *Lemna minor* “Blarney” clone within a multitiered duckweed cultivation system under the four different light treatments (T1, T2, T3, and T4; see Table 1). The dashed lines indicate the initial concentration of TN and TP (i.e., Day 0). Calculated median update values (mg m^−2^ d^−1^) are shown for TN and TP at each light treatment (**C**,**D**). Interquartile ranges (IQR), maximum and minimum IQR values, and outliers (○) are also shown, while × denotes mean values. Shared letters indicate statistical similarity among pairwise comparisons for each panel (a, b or c; *p* > 0.05).

Total nitrogen content (mg L^−1^) of the cultivation medium significantly decreased over the duration of the experiment (GLM: χ^2^ = 63.562, df = 7; *p* < 0.0001; Figure 5A). Medium nitrogen content was significantly affected by day and light regime (both *p* < 0.0001). An interactive effect was not apparent (*p* = 0.06). Medium nitrogen content on Day 7 was significantly lower than the total nitrogen present on Day 0 (all *p* < 0.001).

Total phosphorous content (mg L^−1^) of the cultivation medium significantly decreased over the duration of the experiment (GLM: χ^2^ = 75.789, df = 7; *p* < 0.0001; Figure 5B). Medium phosphorus content was significantly affected by day (*p* < 0.0001), but not by light regime (*p* > 0.05). However, an interactive effect was detected between day and light treatment (*p* < 0.0001). Medium phosphorous content was significantly reduced on Day 7 compared to Day 0 levels in all cases (all *p* < 0.05).

Calculated total nitrogen removal rates (mg TN m^−2^ d^−1^) significantly differed among light regimes (LM: χ^2^ = 11.932, df = 3; *p* < 0.01; Figure 5C). Nitrogen removal rates were significantly reduced for T4 exposed duckweed compared to duckweed maintained at T1 (*p* < 0.01; Figure 5C). In turn, total nitrogen removal rates did not among the other light treatments (all *p* > 0.05). Calculated total phosphorous removal (mg TP m^−2^ d^−1^) did not differ among light regimes (LM: χ^2^ = 3.0671, df = 3; *p* > 0.05; Figure 5D).

## 4. Discussion

### 4.1. Lemna Minor Clones

Although no difference in duckweed growth was detected for the four *L. minor* clones when cultivated on an optimised medium under the same light regime, the “Blarney” clone tended to perform better than other clones when maintained on the VSEP ADLF-pig slurry. In particular, the axenic “Blarney” grew relatively better on a 10% solution of the VSEP ADLF-pig slurry in comparison to the reduced RGRs shown by other clones. The three non-axenic clones collected from the field may have displayed reduced growth due to inherently lower RGRs caused by sub-optimal field conditions, such that the clones may have been deficient of certain micronutrients or otherwise experienced sub-optimal conditions in the field. Although non-axenic cultures were maintained on half-strength Hutner’s medium under laboratory conditions for 2–3 weeks before experimentation, this may have been an insufficient acclimation period to allow for the clones to reach their optimal RGR in advance of experimentation [7]. Future research should consider duckweed acclimation times with a view to optimise RGR of clones prior to experimentation. Although it appears an axenic clone best tolerated the sub-optimal conditions of wastewater cultivation, the present study lends further support to the premise of differing environmental tolerances among duckweed clones obtained from a single species given long-term, multi-generational effects of localised eco-physiological interactions or laboratory conditions [7]. This demonstrates the need to test numerous clones and wastewater pairings to determine optimal duckweed–wastewater pairings for improved system performance with the aim of optimising either remediation capacity or biomass composition [17].

### 4.2. VSEP ADLF-Pig Slurry Dilutions

The dilution of the liquid waste impacted duckweed growth, with greater dilution promoting increased growth. The assessment of a 100% (i.e., non-diluted) VSEP ADLF-pig slurry was omitted as a preliminary assessment determined the cultivation on 100% VSEP ADLF-pig slurry resulted in complete mortality of *L. minor* clones. Although most physicochemical parameters of VSEP ADLF-pig slurry are within known tolerance limits for duckweed, some parameters are beyond the optimal range (see Table 3). In particular, undiluted VSEP ADLF-pig slurry has a relatively high salinity, with key parameters being 2–3 times the maximal of the optimal range (i.e., sodium and chloride; 424 mg L^−1^ and 1319 mg L^−1^, respectively) as determined by Walsh et al. [36]. A seemingly more favourable physicochemical composition can be achieved through dilution of VSEP ADLF-pig slurry. These data for VSEP ADLF-pig slurry correspond to data presented in the literature for other wastewaters (e.g., [42,43]) with dilution likely reducing toxicity, salinity, total solids, initial microbial load, polysaccharides, and proteins found in some wastewaters. In turn, over dilution of wastewater may result in reduced macro- and micronutrient availability, along with a burdensome increase on overall wastewater volume.

### 4.3. Light Regimes: Lemna Minor Cultivation on an Optimised Medium

Higher light intensity did not tend to yield a significant increase in RGR for *L. minor* clones cultivated on half-strength Hutner’s medium. This is consistent with previous studies that indicate *L. minor* does not readily take advantage of high-intensity light conditions that have been found to promote growth of other Lemnaceae (e.g., *L. minuta* [44]). In turn, *L. minor* has shown to more rapidly grow under medium to high light intensities in comparison to *Spirodela polyrhiza*, but with reduced growth at lower light intensity [45]. Duckweed maintained at a 16:8 h light/dark photoperiod tended to show greater RGRs than those kept at 24 h of light, although this was not always statistically apparent. Nevertheless, it appears the photoperiod of 24:0 h light/dark is less than optimal for duckweed cultivation when compared to 16 h of light followed by 8 h of dark. This contrast with findings for cultivation of *Lemna aequinoctialis* 6000 on half-strength Schenk–Hildebrandt medium, whereby Yin et al. [46] demonstrated 24:0 light/dark photoperiod enhanced growth rates, especially under high light intensities. Further, *Landoltia punctata* grown on clean, nutrient-rich laboratory medium was generally observed to grow equally well under different photoperiods (i.e., 12:12, 16:8, 24:0 h light/dark [24]). Interestingly, RGRs of *L. minor* clones grown at similar light intensities grew best under a 16:8 h light/dark photoperiod despite having a lower DLI (i.e., T3). For *L. minor* clones grown on half-strength Hutner’s, DLI did not appear to effect on growth.

### 4.4. Light Regimes: Lemna Minor Cultivation on Wastewater

For these typical laboratory-scale assessments of duckweed growth on a volume of 250 mL, it appears that the photoperiod rather than the light intensity is more important for duckweed growth. Duckweed exposed to VSEP ADLF-pig slurry under 16 h light and 8 h dark (16:8) tended to grow better than those maintained at 24 h light with 0 h dark (24:0) at both high and low light intensities, despite delivery of similarly paired DLIs. Duckweed grown under low light intensity under a 16:8 photoperiod (T3) showed higher RGR values than duckweed cultivated under high light intensity under the same 16:8 photoperiod (T4). Walsh et al. [19] demonstrated the interactive effects of light intensity and wastewater composition on duckweed growth, showing light intensities above 50 µmol m^−2^ s^−1^ did not promote additional duckweed growth or increased remediation. This may be due to several factors such as lack of initial acclimation to growth on a wastewater, change in light intensity and photoperiod, the physicochemical composition of the wastewater, or due to changes in the uptake mechanisms of certain nutrients (e.g., bioavailability of nutrients) [7]. Higher light intensity can amplify symptoms of stress in plants that are not growing optimally; the xanthophyll cycle requires time to upregulate, allowing for the plant to disperse extra energy, preventing the production of reactive oxygen species [47,48]. As experiments in this study were conducted in seven-day periods, plants may not have had sufficient time to acclimate to new growth conditions. Long-term experiments are required to determine if the stress is related to the acclimation or if the negative effects would be exacerbated leading to a further reduction in growth. Parameters such as chlorophyll *a* fluorescence would give a greater indication of photosynthetic health under different photoperiods and light intensities [49]. This is further confirmed by duckweed clones cultivated on the different light treatments on an optimised medium, which showed no negative effect on RGR at higher light intensities with a 16:8 photoperiod.

### 4.5. Larger Scale Indoor Duckweed Cultivation

Optimised duckweed growth appears to require a period of darkness within the light regime. While dark periods reduce energy costs and appear to promote overall duckweed growth on both optimal and sub-optimal media, system design and operation will need to support efficiency and favourable economic outcomes. Large volumes of wastewater are produced continuously or in batches in many agri-food industries, with minimisation of volumes stored and storage durations being desirable. As a result, duckweed systems may have to be designed for either a continuous or large influx of untreated wastewater. Although plants continue to take up nutrients during dark periods [50], the effectiveness of remediation in relation to photoperiod and intensity will require exploration. Rather than longer light periods, extended dark periods could be used to minimise running costs but support overall growth and remediation. Similarly, daily light regimes other than distinct single light and dark periods should also be considered. A shorter and more frequent light-to-dark period should be explored, for example, a continuous cycle of 6 h light and 6 h dark. In turn, cultivation systems could be designed to ensure cultivation tanks and evenly distributed among light and dark periods at any one time, i.e., sperate cultivation tanks could be exposed to alternating lighting periods to ensure half the system receives light while the other half is placed under dark conditions with a view to maximising overall system efficiency.

Numerous studies have sought to identify optimal light intensity for duckweed cultivation, with comparatively less consideration of photoperiod effects [18]. In most cases, light regimes have tended to be explored using small, static laboratory-scale assessment (100–1000 mL), typically using clean laboratory-grade media. Few studies have considered application relevant assessments through testing of duckweed response to light regimes for plants grown on sub-optimal wastewaters (but see Walsh et al. [19]), for different clones within a species, and at large, industry-relevant scales. The present study demonstrates the importance of duckweed–wastewater pairings for optimal growth, remediation, and biomass composition. Agri-feed industries will be required to determine the primary objective of duckweed-based wastewater treatment systems and therefore define optimal pairings for the desired results. Given cultivation medium can influence selection of optimal light intensity and photoperiod, basic assessments should be conducted in a pilot-scale cultivation system that facilitates realistic conditions to ensure reliability and predictability in duckweed growth and wastewater treatment. Other operational parameters such as duckweed crop densities, harvesting cycles, and system residence time also require consideration in tandem with light regimes.

### 4.6. Application by Industry

The adoption of either indoor or outdoor duckweed remediation systems by the agri-food industry is dependent, in part, on the system’s ability to cater for large volumes of wastewaters, to reduce wastewater storage volumes and times, along with maximised remediation and biomass production while minimising costs. Duckweed-based systems provide a double benefit in terms of the production of a protein-rich plant biomass, but also by removing nutrients from the water and reducing wastewater treatment costs. Whilst outdoor duckweed cultivation systems can be scaled to a greater extent (i.e., large ponds), duckweed growth occurs only on the surface of water. Large ponds can hold significant volumes of wastewater; however, this would greatly increase the residence time and leave the system vulnerable to pests and pathogens such as algal blooms. Indoor based cultivation reduces residence time providing cleaned water and a high protein plant biomass daily. Economic and practical considerations frequently underpin industry expectations for continuous cropping of duckweed. However, limited data are available for the cost of alternative treatment processes for agri-food wastewaters [51]—including duckweed-based remediation. In many industry settings, extended growth cycles whereby wastewater is continuously added to duckweed cultivation tanks with sustained daily nutrient uptake and biomass production of duckweed are operationally desirable. Advances in lighting technologies such as renewable energy sources and LED lighting will undoubtedly moderate running costs. However, duckweed systems will need to be both economically and operationally efficient to entice adoption by the agri-food industry. When grown indoors, sufficient light is required for optimal duckweed growth and thus nutrient recovery. Providing artificial light via efficient LED systems can reduce lighting costs significantly. Industry must tailor systems to maximise return based on the value of wastewater treatment or the subsequent valorisation through the production of a high-value plant biomass. Presently, as an emerging market, the value of harvested duckweed can vary considerably (e.g., EUR 2000–EUR 3000 tonne^−1^ [52]), further complicating valorisation assessments of liquid waste streams by the wider agri-food industry. In turn, agri-food enterprises will also need to consider cost-effectiveness in relation to environmental impact abatement measures (e.g., possible economic and reputational incentives to reduce non-renewable resource and energy use [53]).

Lighting regime selection will be governed by economic trade-offs between cost of energy consumed and value biomass or extent of remediation achieved by the cultivation system. Considering a nominal lighting electrical cost of EUR 0.3226 kWh^−1^ over a seven-day growth, the treatments would range in cost—T1 (16:8, 100 µmol m^−2^ s^−1^) > T2 (24:0, 60 µmol m^−2^ s^−1^) > T3 (16:8 55 µmol m^−2^ s^−1^) > T4 (24:0, 35 µmol m^−2^ s^−1^): EUR 9.81 m^−2^ week^−1^; EUR 8.83 m^−2^ week^−1^; EUR 5.40 m^−2^ week^−1^; EUR 5.15 m^−2^ week^−1^. While duckweed cultivated under a high DLI might slightly increase biomass production when compared to a lower DLI, the savings in electrical cost can outweigh the benefit in biomass production at industrial scale. The increase in biomass under a 16:8 photoperiod is minimal at the higher light intensity compared with the lower intensity; however, it incurs an 83% increase in cost (i.e., EUR 9.81 vs. EUR 5.40). The adoption of duckweed cultivation systems requires economic efficiencies such as increased biomass yields and nutrient uptake rates, as well as higher protein content along with lower operational costs being desirable. Nevertheless, identification of a lighting regime that incurs the greatest return on investment, but not necessarily the lowest setup and operational costs, will govern the approach taken by different industries within the wider agri-food sector. Determination of the biomass composition of different duckweed species and clones and thus value will allow for selection of the optimal photoperiod and light intensity pairing.

## 5. Conclusions

The present study provides a proof of concept for the further development of indoor duckweed cultivation systems. Through a comparative assessment of growth for different duckweed clones on an optimal duckweed medium and agricultural wastewater, along with exploration of the effects of light intensity and photoperiod on indoor duckweed cultivation, this study demonstrates that contrasting media necessitate differing light regimes to optimise duckweed growth. These findings emphasise the need to move beyond small-scale and static assessments of duckweed to those grown at larger pilot-plant and industry-relevant scales, whereby additional cultivation parameters (e.g., flow velocity) can impact duckweed growth.

## Figures and Tables

**Table 1:** Light treatment, intensity, and photoperiod with calculated daily light integral.

Treatment	Light Intensity (µmol m^−2^ s^−1^)	Photoperiod (Light/Dark Hours)	Daily Light Integral (mol m^−2^ d^−1^)
T1	100	16:8	5.76
T2	60	24:0	5.18
T3	55	16:8	3.17
T4	35	24:0	3.02

**Table 2:** Name, source environment, and source location of the four *Lemna minor* clones.

Name	Source Environment	Location
Ballinacurra	Semiurban, pastureland drainage ditch	51°53′53.4″ N, 8°09′36.7″ W
Blarney	Semiurban, pastureland freshwater stream	51°56′25.7″ N, 8°33′49.1″ W
Mitchelstown	Dairy processing wastewater treatment plant	52°16′18.2″ N, 8°16′49.8″ W
Sherkin	Coastal, brackish ephemeral pool	51°28′14.1″ N, 9°24′46.0″ W

**Table 3:** Concentration of compounds and elements present in VSEP ADLF-pig slurry ^a^, as well as the required, tolerated, and optimal concentrations for duckweeds.

Parameters	100% VSEP ADLF-Pig Slurry ^a^	Min. Required ^b^ (mg L^−1^)	Max. Tolerated ^b^ (mg L^−1^)	Optimal Range for Duckweed ^b^ (mg L^−1^)
BOD (mg O_2_ L^−1^)	85.0	ND	ND	ND
COD (mg O_2_ L^−1^)	353	ND	ND	ND
Total solids (mg L^−1^)	6753	ND	ND	ND
Total nitrogen (mg N L^−1^)	151.7	0.07	2101	2.8–350
Ammonia (mg N L^−1^)	42.55	ND	98	20–50 ^c^
Nitrate (mg N L^−1^)	126.1	3	>1000	3–300
Nitrite (mg N L^−1^)	0.02	ND	ND	ND
Total phosphorous (mg P L^−1^)	18.0	0.003	310	0.3–54.2
Orthophosphate (mg P L^−1^)	14.86	0.003	310	0.1–50
Chloride (mg Cl^−^ L^−1^)	1319	0.035	3545	0.035–350
Sulphate (mg SO_4_^2−^ L^−1^)	1822.0	0.32	1924	16–641
Potassium (mg K L^−1^)	1910	1.95	1564	20–782
Sodium (mg Na L^−1^)	424	0	4600	0–230
Calcium (mg Ca L^−1^)	5.07	0.4	1600	8–800
Magnesium (mg Mg L^−1^)	1.88	0.1	800	1.2–240
Iron (mg Fe L^−1^)	0.329	0.06	56	0.06–11
Zinc (mg Zn L^−1^)	0.326	0.04	523	0.13–13
Copper (mg Cu L^−1^)	0.048	0.006	64	0.006–3.8
Manganese (mg Mn L^−1^)	0.045	0.005	55	0.05–5.5
Nickel (mg Ni L^−1^)	0.034	0	1	0–0.1

^a^ VSEP ADLF-pig slurry—liquid fraction of anaerobically digested pig slurry (ADLF-pig slurry) obtained from the vibratory shear enhanced processing (VSEP). Anaerobically digested dairy processing wastewater. ^b^ Walsh et al. [36]. ^c^ Körner et al. [37]. ND—Not determined.

## Data Availability

The original contributions presented in this study are included in the article/Supplementary Material. Further inquiries can be directed to the corresponding author.

## Supplementary Materials

The following supporting information can be downloaded at: https://www.mdpi.com/article/10.3390/plants14030397/s1.
