# Supplementary material for: Varied Growth Media Necessitate Different Light Regimes for Indoor Duckweed Cultivation
Source: Plants (Basel). 2025 Jan 28;14(3):397. doi: 10.3390/plants14030397 (PMC11821151; doi:10.3390/plants14030397)
Supplement: Supplementary file 1 [file plants-14-00397-s001.zip › plants-3428261-supplementary.pdf]

Treatment	rep	strain_full	strain	VSEP (0= h	end_colony	end_frond	alive_frond	end_bio	yield
1	1	Ballinacurr	Bal	0	9	47	47	40.4	35.03
1	2	Ballinacurr	Bal	0	5	57	57	55.8	50.43
1	3	Ballinacurr	Bal	0	6	38	38	30.9	25.53
1	1	Blarney	Blar	0	7	35	35	25	19.33
1	2	Blarney	Blar	0	8	68	68	80.8	75.13
1	3	Blarney	Blar	0	6	41	41	31.1	25.43
1	1	Mitchelstov	Mitch	0	14	41	37	34.2	25.57
1	2	Mitchelstov	Mitch	0	11	45	45	44.5	35.87
1	3	Mitchelstov	Mitch	0	13	54	54	79.2	70.57
1	1	Sherkin	Sher	0	10	48	48	54.4	47.9
1	2	Sherkin	Sher	0	3	36	36	49.4	42.9
1	3	Sherkin	Sher	0	4	36	36	34.7	28.2
1	1	Ballinacurr	Bal	10	10	56	56	47.9	42.53
1	2	Ballinacurr	Bal	10	7	20	18	11.1	5.73
1	3	Ballinacurr	Bal	10	10	29	26	17	11.63
2	1	Ballinacurr	Bal	10	4	25	25	17.5	12.13
2	2	Ballinacurr	Bal	10	11	30	30	16.5	11.13
2	3	Ballinacurr	Bal	10	3	22	22	14	8.63
3	1	Ballinacurr	Bal	10	10	52	50	37.8	32.43
3	2	Ballinacurr	Bal	10	6	43	43	31.4	26.03
3	3	Ballinacurr	Bal	10	6	29	29	18.7	13.33
4	1	Ballinacurr	Bal	10	8	33	33	25	19.63
4	2	Ballinacurr	Bal	10	6	33	33	21.2	15.83
4	3	Ballinacurr	Bal	10	7	28	28	17.3	11.93
1	1	Ballinacurr	Bal	25	4	26	25	12.8	7.43
1	2	Ballinacurr	Bal	25	5	29	28	16.8	11.43
1	3	Ballinacurr	Bal	25	4	29	29	18.5	13.13
2	1	Ballinacurr	Bal	25	3	23	23	10.4	5.03
2	2	Ballinacurr	Bal	25	6	29	29	16.2	10.83
2	3	Ballinacurr	Bal	25	4	28	28	14.1	8.73
3	1	Ballinacurr	Bal	25	6	32	32	15.1	9.73
3	2	Ballinacurr	Bal	25	5	31	31	18.3	12.93
3	3	Ballinacurr	Bal	25	11	60	60	56.5	51.13
4	1	Ballinacurr	Bal	25	3	26	26	12.1	6.73
4	2	Ballinacurr	Bal	25	7	31	30	17.4	12.03
4	3	Ballinacurr	Bal	25	3	22	22	14.7	9.33
1	1	Ballinacurr	Bal	50	3	15	15	8.9	3.53
1	2	Ballinacurr	Bal	50	3	16	16	9.8	4.43
1	3	Ballinacurr	Bal	50	4	17	17	12.2	6.83
2	1	Ballinacurr	Bal	50	4	14	14	7.8	2.43
2	2	Ballinacurr	Bal	50	3	13	12	6.7	1.33
2	3	Ballinacurr	Bal	50	7	14	12	7.7	2.33
3	1	Ballinacurr	Bal	50	4	21	20	11.9	6.53

3	2	Ballinacurr	Bal	50	3	22	22	10.5	5.13
3	3	Ballinacurr	Bal	50	5	18	18	10.9	5.53
4	1	Ballinacurr	Bal	50	5	19	18	7.2	1.83
4	2	Ballinacurr	Bal	50	4	20	20	12.1	6.73
4	3	Ballinacurr	Bal	50	4	14	14	7.6	2.23
2	1	Ballinacurr	Bal	0	3	25	25	20.8	15.43
2	2	Ballinacurr	Bal	0	4	32	32	22.2	16.83
2	3	Ballinacurr	Bal	0	4	19	19	13.1	7.73
2	1	Blarney	Blar	0	7	30	30	21.4	15.73
2	2	Blarney	Blar	0	5	43	43	39.7	34.03
2	3	Blarney	Blar	0	3	36	36	25.5	19.83
2	1	Mitchelsto	Mitch	0	13	19	13	13.2	4.57
2	2	Mitchelsto	Mitch	0	8	35	34	36.1	27.47
2	3	Mitchelsto	Mitch	0	6	23	23	21.7	13.07
2	1	Sherkin	Sher	0	8	26	26	22.6	16.1
2	2	Sherkin	Sher	0	3	27	27	22.2	15.7
2	3	Sherkin	Sher	0	5	30	30	24.3	17.8
1	1	Blarney	Blar	10	13	66	66	54.3	48.63
1	2	Blarney	Blar	10	7	62	62	50.6	44.93
1	3	Blarney	Blar	10	5	60	60	75	69.33
2	1	Blarney	Blar	10	6	40	40	27	21.33
2	2	Blarney	Blar	10	5	47	47	42.4	36.73
2	3	Blarney	Blar	10	5	29	29	19.1	13.43
3	1	Blarney	Blar	10	7	54	54	48.2	42.53
3	2	Blarney	Blar	10	10	65	65	57	51.33
3	3	Blarney	Blar	10	10	47	47	59.9	54.23
4	1	Blarney	Blar	10	3	32	32	24.8	19.13
4	2	Blarney	Blar	10	9	42	42	30.9	25.23
4	3	Blarney	Blar	10	10	45	45	41.3	35.63
1	1	Blarney	Blar	25	5	29	29	17.7	12.03
1	2	Blarney	Blar	25	3	40	40	35	29.33
1	3	Blarney	Blar	25	4	29	29	18.4	12.73
2	1	Blarney	Blar	25	5	26	26	16.3	10.63
2	2	Blarney	Blar	25	3	25	25	15.2	9.53
2	3	Blarney	Blar	25	3	31	31	20.3	14.63
3	1	Blarney	Blar	25	3	40	40	31.8	26.13
3	2	Blarney	Blar	25	6	39	39	24.9	19.23
3	3	Blarney	Blar	25	6	38	38	25.8	20.13
4	1	Blarney	Blar	25	4	26	26	15.4	9.73
4	2	Blarney	Blar	25	5	37	36	25.4	19.73
4	3	Blarney	Blar	25	4	24	24	13.9	8.23
1	1	Blarney	Blar	50	5	19	18	13.2	7.53
1	2	Blarney	Blar	50	3	22	22	12.8	7.13
1	3	Blarney	Blar	50	3	19	19	11.4	5.73

2	1	Blarney	Blar	50	3	19	19	8.8	3.13
2	2	Blarney	Blar	50	3	18	18	10.6	4.93
2	3	Blarney	Blar	50	3	12	12	6.9	1.23
3	1	Blarney	Blar	50	4	16	16	9.6	3.93
3	2	Blarney	Blar	50	3	22	22	17.1	11.43
3	3	Blarney	Blar	50	4	20	20	13.1	7.43
4	1	Blarney	Blar	50	4	12	12	9.9	4.23
4	2	Blarney	Blar	50	5	15	15	11.7	6.03
4	3	Blarney	Blar	50	6	12	12	9	3.33
3	1	Ballinacurr	Bal	0	6	32	32	24.2	18.83
3	2	Ballinacurr	Bal	0	5	33	33	24.7	19.33
3	3	Ballinacurr	Bal	0	9	41	41	41.1	35.73
3	1	Blarney	Blar	0	5	46	46	43.6	37.93
3	2	Blarney	Blar	0	16	65	65	72	66.33
3	3	Blarney	Blar	0	10	38	38	27.8	22.13
3	1	Mitchelstov	Mitch	0	12	27	27	25	16.37
3	2	Mitchelstov	Mitch	0	15	51	51	61.1	52.47
3	3	Mitchelstov	Mitch	0	13	45	45	65.8	57.17
3	1	Sherkin	Sher	0	5	45	45	40.1	33.6
3	2	Sherkin	Sher	0	7	39	39	44.6	38.1
3	3	Sherkin	Sher	0	9	34	34	33.5	27
1	1	Mitchelstov	Mitch	10	9	40	40	36.4	27.77
1	2	Mitchelstov	Mitch	10	11	33	32	30.6	21.97
1	3	Mitchelstov	Mitch	10	11	30	29	23.1	14.47
2	1	Mitchelstov	Mitch	10	9	25	25	20.5	11.87
2	2	Mitchelstov	Mitch	10	11	31	31	27.3	18.67
2	3	Mitchelstov	Mitch	10	9	28	28	22.8	14.17
3	1	Mitchelstov	Mitch	10	5	31	31	27.2	18.57
3	2	Mitchelstov	Mitch	10	10	43	43	40.5	31.87
3	3	Mitchelstov	Mitch	10	13	55	55	66.8	58.17
4	1	Mitchelstov	Mitch	10	7	38	38	36	27.37
4	2	Mitchelstov	Mitch	10	10	42	42	43.8	35.17
4	3	Mitchelstov	Mitch	10	19	44	31	31.2	22.57
1	1	Mitchelstov	Mitch	25	4	33	33	32	23.37
1	2	Mitchelstov	Mitch	25	5	34	33	30.2	21.57
1	3	Mitchelstov	Mitch	25	8	30	30	22.4	13.77
2	1	Mitchelstov	Mitch	25	3	25	25	19	10.37
2	2	Mitchelstov	Mitch	25	3	21	21	13.3	4.67
2	3	Mitchelstov	Mitch	25	5	29	28	23.2	14.57
3	1	Mitchelstov	Mitch	25	6	42	42	44.5	35.87
3	2	Mitchelstov	Mitch	25	9	32	32	28.9	20.27
3	3	Mitchelstov	Mitch	25	7	28	28	25.4	16.77
4	1	Mitchelstov	Mitch	25	4	30	30	23.3	14.67
4	2	Mitchelstov	Mitch	25	7	30	30	23.4	14.77

4	3	Mitchelsto	Mitch	25	8	20	17	12.9	4.27
1	1	Mitchelsto	Mitch	50	5	21	21	15.3	6.67
1	2	Mitchelsto	Mitch	50	6	22	22	14.6	5.97
1	3	Mitchelsto	Mitch	50	4	15	15	13.4	4.77
2	1	Mitchelsto	Mitch	50	3	15	15	9.1	0.47
2	2	Mitchelsto	Mitch	50	7	16	15	12	3.37
2	3	Mitchelsto	Mitch	50	6	12	12	7.5	0.000001
3	1	Mitchelsto	Mitch	50	5	20	19	12	3.37
3	2	Mitchelsto	Mitch	50	7	20	20	14.1	5.47
3	3	Mitchelsto	Mitch	50	4	18	18	16.4	7.77
4	1	Mitchelsto	Mitch	50	6	15	15	8.7	0.07
4	2	Mitchelsto	Mitch	50	7	18	16	11.1	2.47
4	3	Mitchelsto	Mitch	50	5	16	15	11.3	2.67
4	1	Ballinacurr	Bal	0	5	25	25	15.5	10.13
4	2	Ballinacurr	Bal	0	10	41	41	36.3	30.93
4	3	Ballinacurr	Bal	0	8	31	31	22.7	17.33
4	1	Blarney	Blar	0	5	32	32	18.7	13.03
4	2	Blarney	Blar	0	11	46	46	36.9	31.23
4	3	Blarney	Blar	0	10	38	38	39.5	33.83
4	1	Mitchelsto	Mitch	0	9	31	31	22.7	14.07
4	2	Mitchelsto	Mitch	0	11	33	33	40.7	32.07
4	3	Mitchelsto	Mitch	0	10	30	29	28.8	20.17
4	1	Sherkin	Sher	0	4	36	36	30.9	24.4
4	2	Sherkin	Sher	0	5	35	35	35.6	29.1
4	3	Sherkin	Sher	0	5	34	34	37.6	31.1
1	1	Sherkin	Sher	10	9	37	37	26.4	19.9
1	2	Sherkin	Sher	10	8	42	42	24.6	18.1
1	3	Sherkin	Sher	10	5	43	43	32.1	25.6
2	1	Sherkin	Sher	10	5	29	29	22.9	16.4
2	2	Sherkin	Sher	10	12	40	40	28.2	21.7
2	3	Sherkin	Sher	10	6	28	28	20	13.5
3	1	Sherkin	Sher	10	5	55	55	50.1	43.6
3	2	Sherkin	Sher	10	9	42	42	45.5	39
3	3	Sherkin	Sher	10	7	41	41	30.6	24.1
4	1	Sherkin	Sher	10	6	33	33	23.7	17.2
4	2	Sherkin	Sher	10	11	44	44	39.1	32.6
4	3	Sherkin	Sher	10	7	25	23	14.3	7.8
1	1	Sherkin	Sher	25	6	30	30	23.7	17.2
1	2	Sherkin	Sher	25	5	39	39	28.8	22.3
1	3	Sherkin	Sher	25	5	28	28	16.1	9.6
2	1	Sherkin	Sher	25	5	25	25	14.2	7.7
2	2	Sherkin	Sher	25	5	27	26	17.8	11.3
2	3	Sherkin	Sher	25	5	24	24	16.9	10.4
3	1	Sherkin	Sher	25	6	50	47	45.2	38.7

3	2	Sherkin	Sher	25	6	31	31	17.1	10.6
3	3	Sherkin	Sher	25	3	27	27	23.3	16.8
4	1	Sherkin	Sher	25	6	35	35	23.1	16.6
4	2	Sherkin	Sher	25	7	22	22	13.7	7.2
4	3	Sherkin	Sher	25	6	28	28	20.8	14.3
1	1	Sherkin	Sher	50	7	24	24	12.6	6.1
1	2	Sherkin	Sher	50	5	21	21	10.8	4.3
1	3	Sherkin	Sher	50	3	18	18	10.4	3.9
2	1	Sherkin	Sher	50	3	17	17	10.5	4
2	2	Sherkin	Sher	50	4	17	17	10.3	3.8
2	3	Sherkin	Sher	50	4	16	15	9.6	3.1
3	1	Sherkin	Sher	50	5	25	25	14	7.5
3	2	Sherkin	Sher	50	3	15	15	8.3	1.8
3	3	Sherkin	Sher	50	5	22	22	12.8	6.3
4	1	Sherkin	Sher	50	3	19	19	11.3	4.8
4	2	Sherkin	Sher	50	3	12	12	7.6	1.1
4	3	Sherkin	Sher	50	4	19	19	10.8	4.3

RGR
0.288286
0.334421
0.24999
0.211955
0.379541
0.243146
0.196712
0.234321
0.316676
0.303509
0.289735
0.239277
0.312613
0.103731
0.164626
0.168768
0.160362
0.13689
0.278783
0.252283
0.178242
0.219721
0.196168
0.167126
0.124088
0.162936
0.176706
0.094425
0.15774
0.137907
0.147695
0.175153
0.336202
0.116054
0.167949
0.14386
0.072175
0.085936
0.11723
0.053328
0.031611
0.051485
0.113673

0.095792
0.101134
0.041893
0.116054
0.049617
0.193446
0.202752
0.127398
0.189743
0.278023
0.214784
0.06071
0.204435
0.131724
0.178021
0.17547
0.188382
0.322762
0.31268
0.3689
0.22295
0.287423
0.1735
0.305739
0.329695
0.336784
0.210808
0.242224
0.283668
0.162625
0.260023
0.168166
0.150854
0.140872
0.182205
0.246325
0.211383
0.216455
0.14274
0.214223
0.1281
0.120718
0.116322
0.099775

0.062795
0.089381
0.028047
0.075225
0.157698
0.119632
0.079621
0.103486
0.066005
0.215075
0.217996
0.29074
0.29141
0.363068
0.227121
0.151947
0.27961
0.290196
0.259939
0.275133
0.234249
0.205618
0.180822
0.140655
0.123597
0.16452
0.138788
0.163996
0.220865
0.292351
0.204039
0.232056
0.183596
0.187213
0.178942
0.136259
0.112742
0.061789
0.141273
0.234321
0.172657
0.154215
0.141887
0.142499

0.057426
0.081801
0.075111
0.062859
0.007576
0.047095
1E-13
0.047095
0.070133
0.09172
0.001154
0.035957
0.038508
0.15143
0.272999
0.205934
0.170476
0.267575
0.277302
0.13816
0.221569
0.172162
0.222708
0.242935
0.250743
0.200223
0.190135
0.228151
0.179905
0.209646
0.160561
0.291746
0.277987
0.221314
0.18481
0.256331
0.112637
0.18481
0.212653
0.129574
0.111634
0.143914
0.136502
0.277042

0.138182
0.182379
0.181147
0.106513
0.166164
0.094556
0.072535
0.067143
0.06851
0.065763
0.055709
0.109608
0.034922
0.096806
0.079
0.022335
0.072535
